# Arthroscopic observation was useful to detect loosening of the femoral component of unicompartmental knee arthroplasty in a recurrent hemoarthrosis

**DOI:** 10.1186/1758-2555-4-8

**Published:** 2012-02-21

**Authors:** Kotaro Yamakado, Hitoshi Arakawa, Seigaku Hayashi

**Affiliations:** 1 Department of Orthopaedics, Fukui General Hospital, 58-16-1 Egami, Fukui, Fukui 9108561, Japan; 2 Arakawa Orthopaedic Clinic, Fukui, Japan

## Abstract

A case of recurrent hemarthrosis of the knee after a mobile-bearing unicompartmental knee arthroplasty (UKA; Oxford UKA) is described. A 58-year-old man met with a road traffic accident 10 months after UKA. He developed anteromedial pain and hemarthrosis of the knee joint 1 month after the accident, which required multiple aspirations. Physical examination showed no instability. Plain radiograph revealed no signs of loosening. All laboratory data, including bleeding and coagulation times, were within normal limits. Diagnostic arthroscopy demonstrated loosening of the femoral component. Any intraarticular pathology other than nonspecific synovitis was ruled out. The loose femoral component and polyethylene meniscal bearing were revised. Since then, hemarthrosis has not recurred.

## Background

Recurrent hemarthrosis after knee arthroplasty is a rare complication. The occurrence is presumed to be less than 1% in total knee arthroplasty (TKA) [1-9], and it is merely reported after UKA [10,11]. The reported causes of hemarthrosis after TKA are impingement of the fat pad or hypertrophic vascular mass of the synovium, femoral flare eroding through an atherosclerotic superior lateral genicular artery, and pigmented villonodular synovitis [1-9]. The reported lesions in nonprosthetic knees are anterior cruciate ligament tears, major meniscus tears, osteochondral fractures, posterior cruciate ligament tears, and coagulation disorder [12-17].

We report a case of recurrent hemarthrosis after UKA caused by loosening of the femoral component.

### Case report

**Figure 1:**
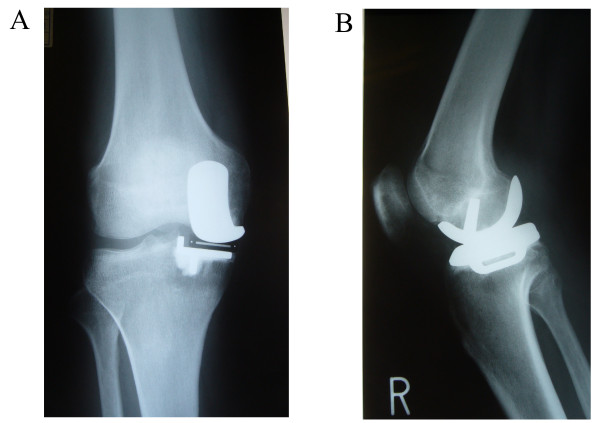
**A: anteroposterior, B: lateral plain radiographs**. No formal signs of loosening such as radolucent lines or osteolysis.

A 58-year-old man met with a road traffic accident 10 months after a mobile-bearing unicompartmental knee arthroplasty (UKA, Oxford UKA; Biomet, Swindon, United Kingdom). His car was struck from the side at an intersection. He could walk on his legs; however, he complained a bruise on the anterior knee. The outcome of the arthroplasty was good with radiological evidence of well-fixed implants before the accident. However, he developed pain and hemarthrosis of the knee joint 1 month after the accident, which subsequently required multiple aspirations of blood (30-50 ml) at an interval of 3-12 days. The range of motion was normal (0/140°), but the patient complained of anteromedial knee pain. Physical examination showed no instability. Plain radiograph revealed no signs of loosening such as radiolucent lines or osteolysis (Figure 1). A small bony gap anterior to the femoral component and a small posterior overhanging were noted in the lateral view of x-ray, however, there was no change compared with the immediate post operative radiographs. All laboratory data, including bleeding and coagulation times, C-reactive protein, ESR were within normal limits. Diagnostic arthroscopy demonstrated loosening of the femoral component and a stable tibial metal tray by palpation with an arthroscopic probe, showing a space between the femoral bone surface and the femoral component (Additional file 1: video, http://www.smarttjournal.com/imedia/1188539294566234/supp1.mpeg). Any other intra-articular pathology including anterior cruciate ligament tears, meniscus tears, osteochondral fractures, posterior cruciate ligament tears, vascular blush other than nonspecific mild synovitis, was ruled out. Synovial tissue and jont fluid was taken as a bacterial culture and was of negative results. After completion of the arthroscopy, an anterior midline mini-arthrotomy incision measuring approximately 5 cm was made through the previous scar. The loose femoral component and polyethylene meniscal bearing were revised. At the bone-prosthesis interface, the cement was primarily intact around the peg, with the loosening occurring at the bone-cement interface. No membranous tissue was observed on the backside of the femoral component and on the cutting surface of the femoral condyle. Since then, hemarthrosis has not recurred until final follow up at 3 years after the revision surgery and the patient was doing well with Knee Society knee score and function score at the latest follow-up evaluation were 95 and 90 points, respectively.

## Discussion

To our knowledge, this is the first report of recurrent hemarthrosis caused by femoral component loosening after UKA. We suppose that a direct blow on the operated knee caused a micro-crack of the cement between the component and the femoral bone surface, which extended to loosening; at the emergency room, he complained a bruise on the anterior knee, indicating that the femoral component was caught between the dashboard and the femoral bone surface. It appeared that micromotion of the component caused a continuous bleeding from the bone bed, which gave rise to recurrent hemarthrosis in a semi-acute setting. In this case, no membranous tissue was observed on the backside of the femoral component and the patient had been doing well before the accident, indicating the acute/semi-acute nature of the loosening. Any other intra-articular pathology was ruled out by the arthroscopic exploration. Mild infection should also be ruled out at every revision surgeries, however, bacterial culture, blood analysis, and post-operative course suggested that infection was unlikely in our case. The circumstantial evidence points to the trauma as a cause of component loosening. Recurrent hemarthrosis after UKA, in itself, is a very rare complication [10,11]. There are only two cases were reported in English literatures. Maheshwari reported a case of spontaneous hemarthrosis caused by the saphenous branch of the descending genicular artery with a prominent vascular blush, which was successfully treated by coil embolization [10]. Raet reported a case of spontaneous hemarthrosis caused by traumatic rupture of the metal marker wire of an all-polyethylene inlay tibial implant, which caused destruction of the polyethylene surface and a disseminated synovitis [11]. The reported causes of hemarthrosis after TKA or in nonprosthetic knee are cruciate ligament tears, meniscus tears, osteochondral fractures, impingement of the fat pad or hypertrophic vascular mass of the synovium, and pigmented villonodular synovitis [1-9,12-17]. Arthroscopic examination had successfully ruled out these intra-articular pathologies in our case. Loosening of the femoral component after UKA (Oxford UKA) is the second most common cause of revision, and the incidence ranges from 0% to 2.1% [18-21], however, diagnosis of femoral component loosening is difficult [21]. Symptoms related to component loosening are nonspecific and are usually reported as pain. Hence, it is very important to determine this early in the clinical course. There might be very subtle changes on standard anteroposterior or lateral radiographs before osteolysis occurs. Thus, comparison between early post-operative radiographs and present radiographs may show migration of the component. However, it is difficult to distinguish the loosening of femoral component using radiographs because back surface is concave and bonecement interface cannot be seen in radiographs. Moreover, it must be required to delineate the difference between pathological and physiological, non progressive, such radiolucencies which have been shown to be common and not related to loosening. Recently, as an alternative, Monk et al. described a technique using lateral extension and flexion radiographs [21], which might to be a powerful tool to obviate the need for artrhroscopy. We think that the arthroscopy is invasive and should be reserved procedure, however, it would be very worthful if other examination methods failed to specify and/or rule out the component loosening.

## Conclusions

In conclusion, our case showed that loosening of the femoral component might lead to recurrent hemarthrosis and suggested that arthroscopic exploration was thought to be one of useful methods to diagnose and, especially, to rule out other intra-articular pathology.

## Abbreviations

UKA: Unicompartmental knee arthroplasty; TKA: Total knee arthroplasty.

## Competing interests

The authors declare that they have no competing interests.

## Authors' contributions

KY and HA participated in this study and KY drafted the manuscript. SH supervised this study. All authors read and approved the final manuscript.

## Supplementary Material

Additional file 1 **A space between the femoral bone surface and the femoral component is shown**. Click here for file
